# A Directed Molecular Evolution Approach to Improved Immunogenicity of the HIV-1 Envelope Glycoprotein

**DOI:** 10.1371/journal.pone.0020927

**Published:** 2011-06-29

**Authors:** Sean X. Du, Li Xu, Wenge Zhang, Susan Tang, Rebecca I. Boenig, Helen Chen, Ellaine B. Mariano, Michael B. Zwick, Paul W. H. I. Parren, Dennis R. Burton, Terri Wrin, Christos J. Petropoulos, John A. Ballantyne, Michael Chambers, Robert G. Whalen

**Affiliations:** 1 Department of Infectious Diseases, Maxygen, Inc., Redwood City, California, United States of America; 2 Department of Immunology and Microbial Science, The Scripps Research Institute, La Jolla, California, United States of America; 3 Department of Immunology and Microbial Science, and IAVI Neutralizing Antibody Center, The Scripps Research Institute, La Jolla, California, United States of America; 4 Ragon Institute of Massachusetts General Hospital, Massachusetts Institute of Technology and Harvard, Boston, Massachusetts, United States of America; 5 Monogram Biosciences, San Francisco, California, United States of America; 6 Aldevron LLC, Fargo, North Dakota, United States of America; University of Cape Town, South Africa

## Abstract

A prophylactic vaccine is needed to slow the spread of HIV-1 infection. Optimization of the wild-type envelope glycoproteins to create immunogens that can elicit effective neutralizing antibodies is a high priority. Starting with ten genes encoding subtype B HIV-1 gp120 envelope glycoproteins and using *in vitro* homologous DNA recombination, we created chimeric gp120 variants that were screened for their ability to bind neutralizing monoclonal antibodies. Hundreds of variants were identified with novel antigenic phenotypes that exhibit considerable sequence diversity. Immunization of rabbits with these gp120 variants demonstrated that the majority can induce neutralizing antibodies to HIV-1. One novel variant, called ST-008, induced significantly improved neutralizing antibody responses when assayed against a large panel of primary HIV-1 isolates. Further study of various deletion constructs of ST-008 showed that the enhanced immunogenicity results from a combination of effective DNA priming, an enhanced V3-based response, and an improved response to the constant backbone sequences.

## Introduction

A critical objective in the search for a vaccine to HIV-1 is the identification of immunogens that can elicit antibodies capable of neutralizing a broad array of clinically relevant viruses [1]–[3]. The viral envelope glycoprotein (Env) is central to vaccine research since it is the only target for neutralizing antibodies [1], [4], [5]. The Env consists of the gp120 surface glycoprotein and the gp41 transmembrane protein associated in a trimer of gp120-gp41 heterodimers. The existence of broadly neutralizing sera from some HIV-1 infected individuals [1], [6]–[10] and the protection in monkeys by passive transfer of several neutralizing monoclonal antibodies (mAbs) [11]–[16] suggest that if a suitable antibody response to Env can be obtained, then protection from infection will be possible. However, a large clinical trial using a recombinant version of monomeric gp120 failed to provide any evidence of protection [17]. More recently, the combination of a viral vaccine and recombinant protein resulted in limited but significant protection from infection [18]. It is not known which immune responses are responsible for this result.

HIV-1 virus has evolved multiple mechanisms to evade immune surveillance that include extensive glycosylation, hypervariability of amino acid sequences, conformational masking and inaccessibility of conserved sites [1]–[3], [19]. The major challenge to creating an Env-based antibody-inducing vaccine is the identification of conserved neutralizing epitopes that are both immunogenic enough to induce antibodies and accessible on the virus.

Several forms of Env have been evaluated for immunogenicity including gp120 monomers, soluble gp140 oligomers, and Env-containing virus-like particles [17], [20]–[34]. Attempts have been made to delete certain variable regions [35], [36], create hyperglycosylated forms [37], [38], constrain the CD4-binding conformation of the protein [26], [32], and immunize with mixtures of wild-type sequences [33], [34], in the hope of directing the humoral immune response to more conserved epitopes while limiting the immunogenicity of dominant but non-neutralizing epitopes. For gp140-based immunogens, efforts have focused on stabilizing and increasing trimerization to mimic the conformation of the functional Env spikes on HIV-1 virions [21]–[25], [30], [31], [39]. Additionally, computational approaches have been used to deduce ancestral and consensus sequences of the various HIV-1 subtype and group M Env proteins in an effort to overcome sequence diversity [40]–[43]. Some increased potency of the neutralizing antibodies induced by certain Env formats has been claimed; however, the breadth of neutralization is still so limited that an HIV vaccine able to induce sterilizing immunity will likely not be possible without a fundamental breakthrough [1], [2].

Directed molecular evolution is an effective approach for the improvement of protein function, ranging from enzyme activities [44]–[46] to receptor-ligand interactions [47]–[49]. Directed molecular evolution includes a process to create large libraries of genes expressing diverse protein sequences, which are not typically found in nature, and a means to evaluate the novel proteins for the desired functional property. Many methods are available to create sequence diversity and one of the most powerful is *in vitro* DNA recombination of naturally occurring homologous genes [44], which can produce libraries of chimeric protein-coding genes of high functional quality [50]. The homologous recombination method offers the important advantage that the DNA sequences encoding amino acids beneficial to the desired phenotype can be combined into a single gene. Furthermore, if incremental improvements in protein function are identified, then additional rounds of directed molecular evolution can be used to produce further enhancements [46], [51].

Application of directed molecular evolution has led to the identification of several chimeric dengue envelope proteins that are each capable of inducing neutralizing antibodies against all four dengue serotypes [52]. Dupuy *et al* showed that the application of directed molecular evolution can improve the immunogenicity and protective efficacy of the Venezuelan equine encephalitis virus envelope proteins [53]. Here we report the first successful use of this technology to improve the neutralization responses given by HIV-1 Env immunogens. Since evaluation of immunogenicity requires testing in animals and eventually in humans, the practical application of directed molecular evolution to HIV-1 vaccine improvement faces formidable obstacles. In the present study, we have addressed a number of these issues, developed an *in vitro* screening process to down-select candidates for immunization, implemented high-throughput *in vivo* screening, and identified an immunogen capable of inducing a more potent and broader neutralizing response in rabbits.

## Results

### Selection and characterization of parental gp120 sequences

**Figure 1:**
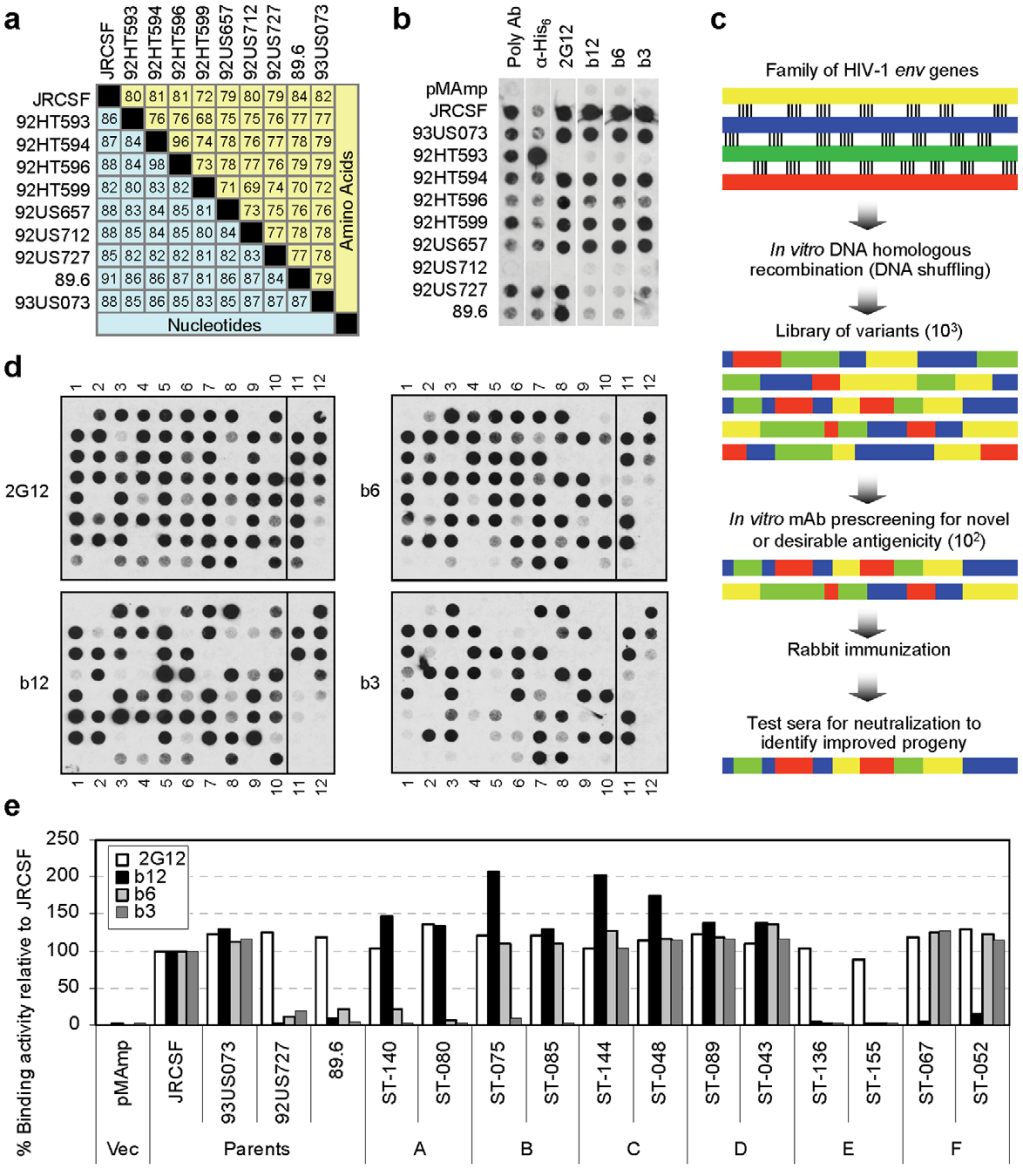
*In vitro* DNA recombination is used to generate gp120 variants with novel antigenic profiles. (**a**) Similarities of parental gp120 sequences. The percent identity for nucleotide sequence comparison is in yellow and that for amino acid comparison in blue. (**b**) Characterization of the parental gp120 proteins. Supernatants obtained from CHO-K1 cells transfected with the indicated parental gp120 were immobilized on dot-blot membranes and analyzed by a mouse polyclonal antiserum (Poly Ab), an anti-His-tag mAb (α-H6), and human anti-gp120 mAbs, 2G12, b12, b6 and b3. (**c**) Schematic illustration of directed molecular evolution of HIV-1 *env* genes. (**d**) Characterization of recombined gp120 variants. Supernatants from transfection of gp120 variants (columns 1–10) and control plasmids (column 11) as well as eight 1∶2 serial dilutions of purified gp120 (column 12) were immobilized on four replicate dot blots and analyzed for reactivity to 2G12, b3, b6 and b12. (**e**) Antigenic profiles of two representative gp120 variants from each of six antigenic categories identified (see Table 1). Binding intensities were quantified from dot blots and normalized against the internal JRCSF control.

The parental gene sequences selected for *in vitro* homologous DNA recombination should be similar enough to ensure extensive recombination while introducing adequate sequence diversity. We chose 10 *env* genes from subtype B HIV-1 (Table S1). Pairwise comparisons of the encoded gp120 amino acid sequences show similarities between 68 to 96%, which is typical of the diversity found within a subtype (Figure 1a). The nucleic acid sequences are 80–98% identical (Figure 1a), indicating that there are many regions where crossovers can occur during *in vitro* DNA recombination.

We transfected plasmids expressing each of the ten parental gp120 genes into CHO-K1 cells and analyzed the resulting secreted proteins on dot-blots for their ability to bind an anti-His-tag mAb, a polyclonal serum against gp120, and the human anti-gp120 mAbs 2G12, b3, b6, and b12. The 2G12 and b12 mAbs can neutralize many strains of HIV-1 and are therefore considered broadly neutralizing. The b3 and b6 mAbs compete with b12 for the CD4 binding site (CD4BS) on gp120 but do not neutralize typical viral isolates.

Figure 1b shows that all of the parents except 92HT593 and 92US712 produced gp120 proteins that were readily detected with the polyclonal antiserum, the anti-His-tag mAb, and at least one of the four human mAbs. The 92HT593 and 92US712 gp120 coding sequences were found to have internal stop codons, probably introduced during PCR amplification steps. The expression levels of the remaining eight parental gp120 proteins were similar as judged by the anti-His-tag signals and their reactivity to 2G12 (Figure 1b). The differences in binding of the polyclonal anti-Env serum are likely due to the sequence differences of up to 32% among these gp120 proteins. The reactivities with the three anti-CD4BS mAbs (b3, b6 and b12) appeared to segregate into two groups, i.e., either equally strong (JRCSF, 93US073, 92HT594, 92HT599, 92US657) or equally weak (92HT596, 92US727, 89.6) (Figure 1b).

### Directed molecular evolution of gp120 identifies antigenic diversity

We used *in vitro* DNA recombination of parental *env* genes to create libraries of chimeric coding sequences expressing gp120 variants. The four human anti-gp120 mAbs were used to systematically screen the variants for different antigenic phenotypes. We categorized the binding activities of individual gp120 variants with particular attention to variants that bind the neutralizing mAbs but not the non-neutralizing mAbs. We hypothesized that such variants would be superior immunogens with respect to their ability to induce neutralizing antibodies upon immunization in animals. The parental gp120 proteins do not exhibit this specific antigenic phenotype.

Two libraries, L4-A and L4-B, were constructed for this work using *in vitro* DNA recombination as illustrated schematically in Figure 1c. Library L4-A includes all ten parental genes to maximize sequence diversity, whereas L4-B omits the 92HT593 and 92US712 gp120 to avoid the internal stop codons. In a first tier of screening, plasmid DNAs from 1000 L4-A clones and 2520 L4-B clones were individually transfected into CHO-K1 cells and the secreted proteins were initially evaluated for binding to b12 and a pool of b3 and b6 on dot blots. A combined total of 249 plasmids produced variants with positive binding to either reagent and were re-transfected into CHO-K1 cells for a second tier of screening to quantify binding to all four mAbs. Figure 1d shows a representative set of dot-blot images for eighty variants from the second tier of screening.

After assessing the reactivity with these mAbs, a total of 207 clones from both libraries were categorized into 6 different antigenic phenotypes (A through F) based on the binding ratios of b12/b6 and b12/b3, as shown in detail in Table 1. The mAb-binding characteristics of two representative variants from each category are shown in Figure 1e. Variants in categories A, B, C and F demonstrated novel patterns of binding to the three anti-CD4BS mAbs that differ from those of the parental gp120 proteins, whose binding patterns fall into categories D and E (Figure 1e).

**Table 1:** Antigenic categories of recombined subtype B gp120 variants.

Category	Relative mAb binding*				Binding Ratios		Representative Phenotype**				No. of Variants***		
	2G12	b12	b6	b3	b12/b6	b12/b3	2G12	b12	b6	b3	Total	*In vivo*	SF162
**A**	Var	Pos	Var	Var	>2.5	>2.5	+++	+++	−	−	33	33	17
**B**	Var	Pos	Var	Var	<2.5	>2.5	+++	+++	+++	−	28	28	13
**C**	Var	Pos	Var	Var	Either = 1.5–2.5		+++	+++	+	+	11	11	5
**D**	Var	Pos	Pos	Pos	0.7–1.5	0.7–1.5	+++	+++	+++	+++	45	10	8
**E**	Var	Neg	Neg	Neg	n/a	n/a	+++	−	−	−	35	8	6
**F**	Var	Var	Either Pos		<0.7 or n/a		+++	−	+++	+++	55	8	3
**Total**											207	98	52

*The cutoff for positive binding is >10% of WT signal; Pos, positive; Neg, negative; Var, varied from negative to positive.

**+++ is ≥100% reactivity relative to that of WT JRCSF; n/a: not applicable.

***The column labeled “*In vivo*” indicates the number of variants from each category selected for rabbit immunization. The column labeled “SF162” indicates the number of variants that induced sera with positive neutralization against SF162 (defined as >50% neutralization at a serum dilution of 1∶7.5).

### Novel antigenic and genetic characteristics of gp120 variants

**Figure 2:**
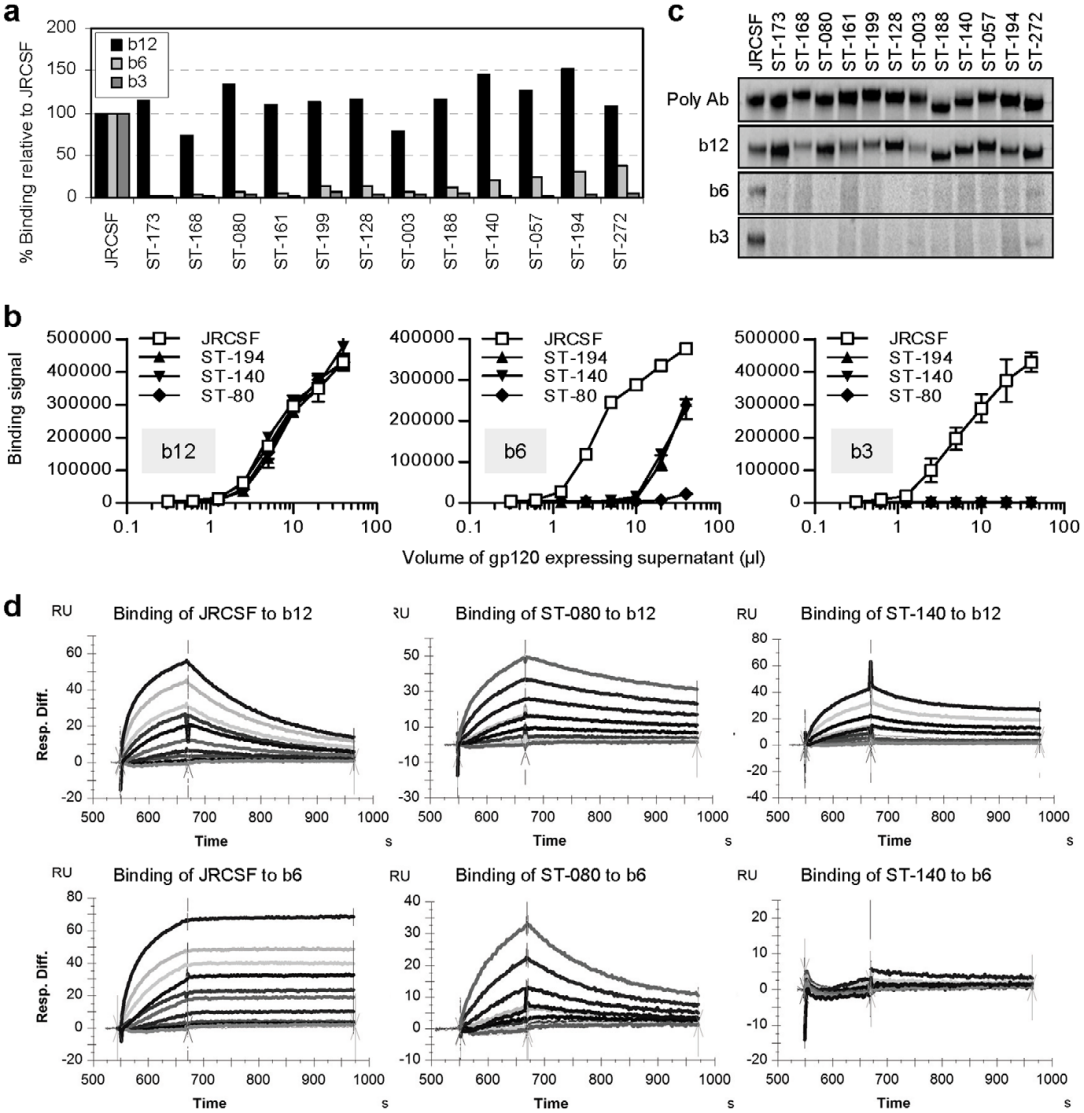
Confirmation of novel antigenicity. (**a**) Antigenic profiles of 12 category A gp120 variants. Data were acquired as in Figure 1e. (**b**) Characterization of category A gp120 with dot blots. Eight 1∶2 serial dilutions of supernatants from triplicate transfection of each indicated gp120 construct were blotted and reacted with b3, b6, b12, and 2G12. Mean and standard deviation of binding intensities at each dilution point are shown. (**c**) Characterization of category A gp120 by immunoprecipitation. Indicated gp120 variants were transiently expressed, metabolically labeled, immunoprecipitated by a mouse polyclonal antiserum (Poly Ab), b12, b6, and b3, and separated on SDS-PAGE. Radiograms of the gels are shown. (**d**) Characterization of category A gp120 by surface plasmon resonance analysis. b6 and b12 were captured on to a CM5 chip immobilized with anti-human antibody and interacted with a range of concentrations of purified gp120 as analyte. Shown are the raw sensor data subtracted by the response of a reference surface for kinetic analysis (Table S2).

The conditions of the high-throughput dot-blot screening method were chosen to allow for reproducible quantitative estimates of mAb binding with a single dilution of the supernatants from transfected cells. To verify that the novel antigenic properties held true over a broad range of concentrations using the same dot-blot assay, we selected three variants with the strongest b12-binding from category A (ST-080, ST-140 and ST-194; see Figure 2a), and performed transient transfections in triplicate using the corresponding plasmids. An 8-point serial dilution of each supernatant was prepared and four replicate blots were made and reacted with b3, b6, and b12. The results confirmed that all three gp120 variants bound b12 as strongly as did JRCSF gp120 but had different b6 binding characteristics, consistent with the screening results obtained using a single dilution (compare Figure 2a and b). Binding to b3 was undetectable for all three variant proteins, representing a greater than 128-fold reduction in the b3-binding activity relative to that given by JRCSF gp120.

Since the dot-blot assay involves immobilization of antigen to a solid-phase nitrocellulose matrix, we also investigated whether the antibody-binding characteristics of the novel variants could be confirmed by immunoprecipitation, which is based on liquid-phase interactions. Twelve category A variants (Figure 2a) were radiolabeled and immunoprecipitated by b3, b6, and b12. Figure 2c shows that all variants bound b12 but not to b3 or b6 in this assay, consistent with the screening results shown in Figure 2a.

To study the basis for the altered antigenicity, we chose two category A gp120 variants, ST-080 and ST-140, and analyzed the kinetics of interaction with mAbs b6 and b12 using surface plasmon resonance. As shown in Figure 2d and Table S2, the wild-type JRCSF gp120 protein binds to b12 with an affinity typical of antigen-antibody interactions (Ka≈10^7^); however, b6 binding affinity is much stronger than that of b12. Compared to JRCSF, the ST-080 and ST-140 gp120 proteins showed 3.4-fold and 2.1-fold higher affinity for b12, respectively, due largely to a decreased off-rate (kd). In contrast, the b6 affinity for both variants was significantly reduced relative to that of JRCSF or completely abolished, representing a >10,000-fold change. These results confirm that the two closely related CD4BS mAb-binding activities can be segregated by use of directed molecular evolution.

**Figure 3:**
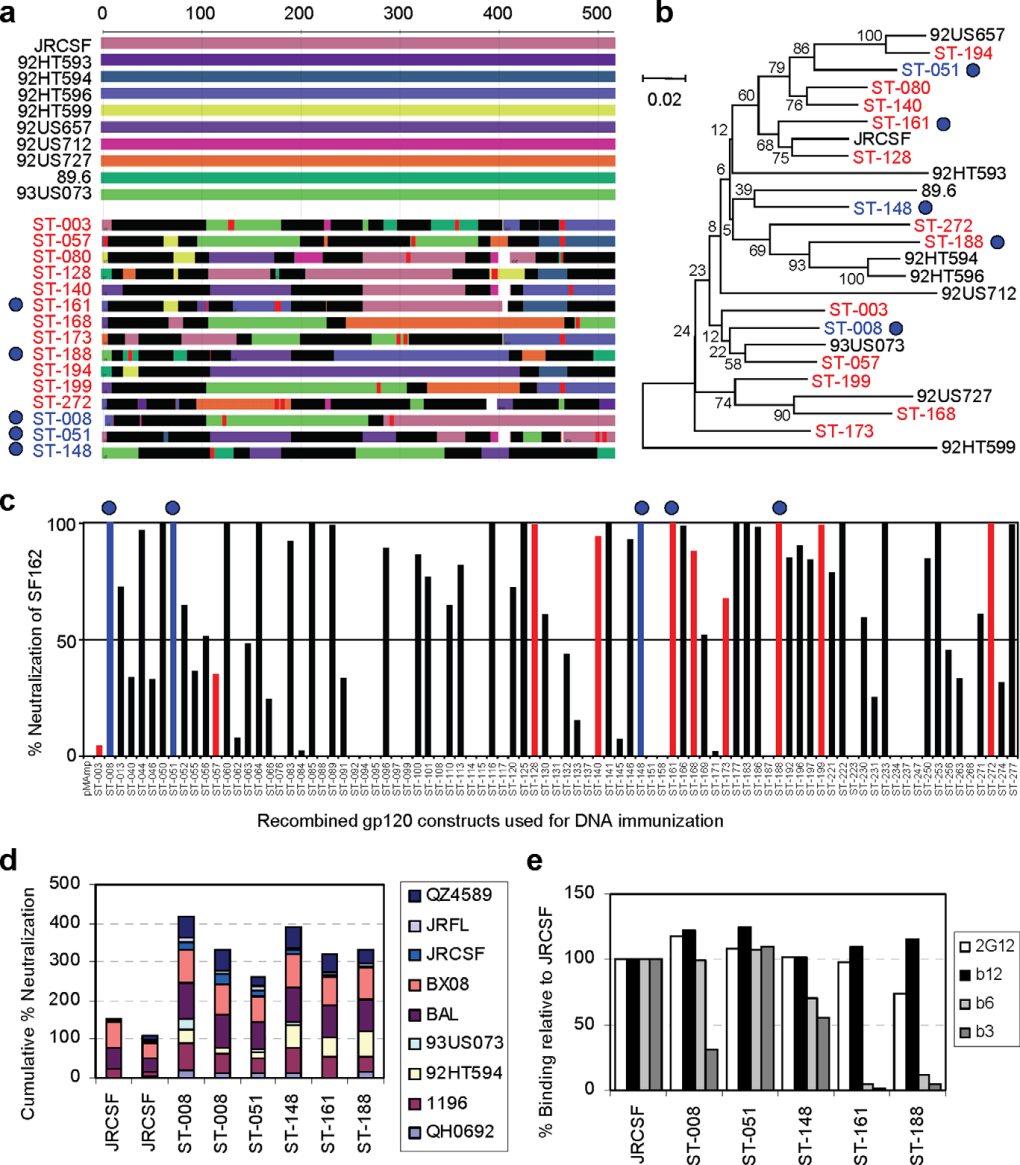
Recombined gp120 variants are genetically diverse and can induce strong neutralization responses. For names shown in (**a**) and (**b**), parents in black; category A gp120 variants in red; non-A variants in blue. Five variants shown in (**d**) and (**e**) are indicated as filled blue circles in (**a**) through (**c**). (**a**) Chimerism of recombined gp120 amino acid sequences. Regions in each variant with sequence identical to each parent have the matching color to the parent: black, sequences identical to more than one parent; red, point mutation; clear, sequences unmatched with any parent. (**b**) Phylogenetic analysis of sequences shown in (**a**). (**c**) Recombined gp120 induced neutralization response to HIV-1 SF-162. Selected gp120 variants (Table 1) were used to immunize two rabbits per clone. Antibodies were purified from terminal bleeds and tested at a dilution equivalent to 1∶7.5 of sera. The result for the better rabbit for each clone is presented. Red and blue bars indicate results by category A and non-A immunogenically improved gp120 shown in (**a**) and (**b**), respectively. (**d**) Direct molecular evolution improved immunogenicity. Antibodies of the 5 improved variants prepared in (**c**) were analyzed against 9 pseudoviruses at a dilution equivalent to 1∶22.5 of the sera. The results for rabbits better than both JRCSF-immunized ones are presented in stack histogram. (**e**) Antigenic profile of the 5 most immunogenic recombined gp120 variants. Data were acquired as in Figure 1e.

To investigate the genetic relatedness of variants that exhibit similar antigenic profiles, we analyzed the chimerism (Figure 3a) of the amino acid sequences of the 12 category A variants studied above. We identified the parental sequences contained in the chimeras and found that each variant had a unique recombination pattern with multiple crossover events (Figure 3a). Phylogenetic analysis further showed that all 12 gp120 variants were distributed relatively evenly throughout the sequence space formed by the 10 wild-type parents without any apparent clustering around individual parents (Figure 3b).

### Neutralization responses induced by gp120 variants

Multiple gp120 proteins from each of the antigenic categories were chosen for immunogenicity studies. The identification of gp120 variants that preferentially bind the broadly neutralizing mAb b12 relative to b3 and b6 allowed us to evaluate the hypothesis that such proteins, which clearly expose the conserved CD4BS epitope, are superior in their ability to induce neutralizing antibodies compared to variants that also bind the non- neutralizing mAbs.

We selected 98 gp120 variants for immunization of rabbits along with five parental gp120 immunogens (JRCSF, 92HT594, 92US657, 93US073, and 89.6). About three-quarters of the 98 variants came from categories A, B and C: they bind the neutralizing mAbs 2G12 and b12 and have novel antigenic profiles compared to parental gp120 proteins. The remaining variants were chosen from categories D, E and F (Table 1). For *in vivo* screening, each gp120-expressing plasmid DNA was injected three times with electroporation into two rabbits at four-week intervals. Four weeks after the third DNA injection, each rabbit received an injection of JRCSF gp120 protein in the expectation that this would boost any cross-reactive antibodies. The sera obtained from each rabbit two weeks after the protein boost (Day 98) were screened for their ability to neutralize the SF162 pseudovirus. At a dilution of 1∶7.5, ∼53% of the variant Env sequences induced antibodies with >50% neutralization (Figure 3c), suggesting that the recombined variants largely retained the immunologically relevant structure of gp120.

There appears to be a weak correlation between antigenic phenotype and the neutralization of SF162 (Figure 3c; Table 1). The percentages of clones that induced >50% neutralization of SF162 were 52%, 46%, 45%, 80%, 75%, and 38% for categories A–F, respectively. Variants from categories D and E (parental antigenic profiles) showed higher positive rates than those from categories A, B, C, and F (novel antigenic profiles), suggesting that evolving away from the parental antigenic phenotypes can influence immunogenicity.

Sera that were positive against SF162 (Figure 3c) were further evaluated using a panel of 9 HIV-1 subtype B pseudoviruses with a wide spectrum of sensitivity to neutralization. Among the five parents tested, JRCSF gp120 consistently outperformed other parents (data not shown). Five gp120 variants (ST-008, ST-051, ST-148, ST-161 and ST-188) had at least one serum that performed better than both sera induced by JRCSF gp120 judging by their cumulative percentage of neutralization against all 9 viruses (Figure 3d). Of these five variants, ST-161 and ST-188 are category A variants, ST-008 is category B, ST-148 is category C, and ST-051 is category D (compare Figure 3e to category definitions in Figure 1e and Table 1). Although these five gp120 variants showed different patterns of binding to the non-neutralizing mAbs b3 and b6, they all bound strongly to the neutralizing mAbs 2G12 and b12 (Figure 3e). The five variants were not related in any obvious way as judged by their chimeric pattern and showed no similarity in their phylogenetic relationships (Figure 3a,b, filled blue circles). These results show that directed molecular evolution can give rise to multiple distinct variants with improved immunogenicity (i.e., increased neutralization potency) compared to the wild-type parental proteins.

### Characterization of the immunogenicity of ST-008

The ST-008 gp120 induced somewhat stronger neutralization activity than the other variants tested. We therefore compared the immunogenicity of the ST-008 and JRCSF gp120 proteins in an experiment using 8 rabbits for each construct. The amino acid sequences of these two proteins are aligned in Figure S1. As before, three electroporation-mediated DNA injections plus one JRCSF protein boost were used. Sera were collected two weeks following the third DNA injection (Day 70) and two weeks after the protein boost (Day 98). All 32 sera were tested for neutralization activity against a panel of 9 subtype B and 6 non-subtype B pseudoviruses that represent a wide spectrum of sensitivity to a broadly neutralizing human plasma [54] (N16) as well as to the broadly neutralizing human mAb b12 (Table 2).

**Table 2:** Neutralization of pseudoviruses by human plasma, b12 and D70 sera shown in Fig. 4A.

Peudo viruses	Subtype	Controls		Comparison of Day 70 sera			
		Human plasma N16(GMT**)	b12(µg/ml)	JRCSF*(GMT**)	ST-008*(GMT**)	FoldIncrease*	*P* value***
SF162	B	12012	0.01	1314	4211	3.2	0.010
NL4-3	B	2128	0.01	89	198	2.2	0.041
BaL	B	1176	0.01	99	172	1.7	0.056
VLGCG4	G	576	0.04	31	50	1.6	0.032
1196	B	411	0.48	41	69	1.7	0.058
QZ4589	B	365	0.03	37	64	1.7	0.028
JRCSF	B	351	0.06	32	101	3.2	0.038
93BR020	F	298	2.08	14	47	3.4	0.005
93MW960	C	261	0.06	14	12	0.9	>0.100
93BR029	F	242	1.67	17	27	1.6	0.039
6535	B	226	0.64	10	41	4.1	0.001
92HT594	B	147	0.02	15	19	1.3	>0.100
QH0692	B	105	0.10	13	20	1.5	0.009
93TH305	E	87	0.71	21	25	1.2	>0.100
92RW020	A	56	1.67	16	12	0.8	>0.100
aMLV	—	<20	>2.50	10	10	1.0	>0.100

*JRCSF & ST-008, immunogens; fold increase, ST-008/JRCSF.

**GMT, geometric mean of IC50 titers for each immunization group.

***Two-tailed homoscedastic t-test using log_10_ (IC50); *n* = 8.

**Figure 4:**
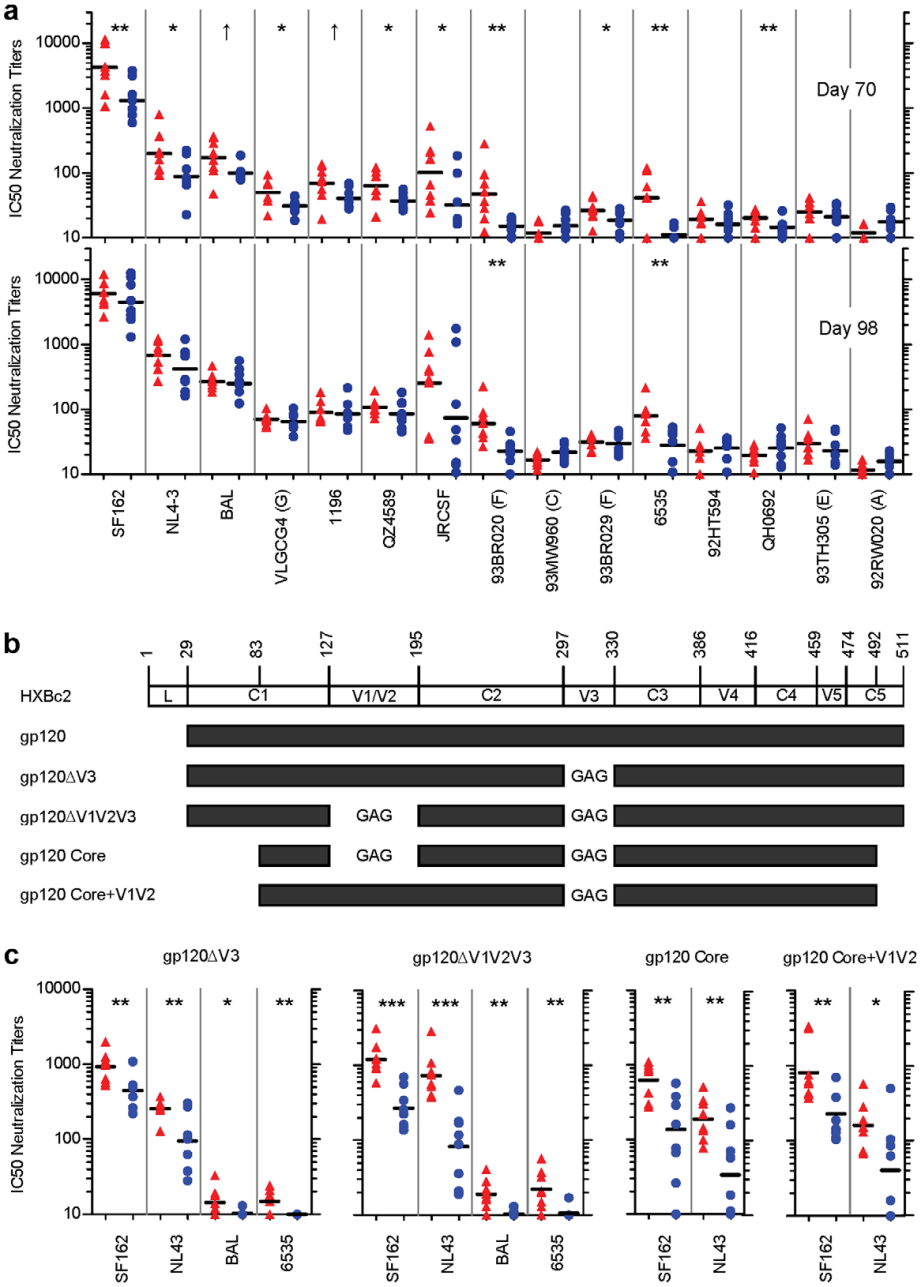
Characterization of the neutralizing antibody responses induced by ST-008. For (**a**) and (**c**), 8 rabbits were immunized with 3 DNA electroporations followed by one protein boost. Sera were evaluated for neutralizing activities against a panel of HIV-1 pseudoviruses. Neutralizing titers (IC50) by ST-008 and JRCSF immunogens are indicated by solid red triangles and blue solid circles, respectively. The geometric mean titer for each group is shown as black horizontal bar. The viruses are presented from left to right in increasing resistance to neutralization to N16 (see Table 2). *** *P*<0.001; ** *P*<0.01; * *P*<0.05; ↑ *P*<0.10 (see *P* values in Table 2 and Table S3). (**a**) Comparison between ST-008 and JRCSF gp120. Rabbits were boosted with JRCSF gp120. Day 70, after 3 DNA injections; Day 98, after protein boost. The subtype of each non-B virus is indicated in parentheses. (**b**) Schematic representation gp120 deletion constructs used in (**c**). (**c**) Comparison of 4 pairs of gp120 deletion constructs made from ST-008 and JRCSF. Rabbits were boosted with homologous proteins. Results of Day 98 sera are shown.

As shown in Figure 4a (upper panel) and Table 2, the neutralization activity of the Day 70 sera from JRCSF-immunized rabbits seemed to track with the neutralization sensitivity of these pseudoviruses to N16 human plasma, with the exception of the JRCSF pseudovirus (autologous neutralization). The Day 70 sera of ST-008-immunized rabbits neutralized all 15 pseudoviruses to varying degrees but with a pattern of neutralization that was different from those given by the JRCSF sera and the N16 plasma. ST-008 was significantly better than JRCSF at inducing neutralizing antibodies for 9 out of 15 pseudoviruses by 1.5- to 4.1-fold (*P*<0.05). On two additional pseudoviruses, BaL and 1196, the neutralizing activities induced by ST-008 trended higher than those of JRCSF by 1.7-fold (*P* = 0.056 and 0.058, respectively). In no instance was neutralization by JRCSF sera significantly greater than that given by ST-008 sera. The superior neutralization activity induced by ST-008 was seen among both the sensitive and more resistant pseudoviruses. The geometric mean titer (GMT) of the ST-008 sera compared to that of the JRCSF sera was 4.1-fold greater for 6535, 3.2-fold greater for JRCSF, and 3.3-fold greater for 93BR020; these three viruses are moderately resistant to neutralization (Table 2).

After the protein boost (Day 98 sera), the differences in neutralization activity induced by ST-008 and JRCSF were less striking (Figure 4a, lower panel). The Day 98 sera of ST-008-immunized rabbits still demonstrated significantly improved neutralization potency on two relatively resistant viruses, 6535 and 93BR020 (*P* = 0.004 and 0.003, respectively). Although not statistically significant, GMT values for ST-008 sera were also 3.4-fold higher against JRCSF and 1.3- to 1.6-fold higher against SF162, NL-43, QZ4587 and 93TH305. These results confirm the immunogenic phenotype of ST-008 observed during *in vivo* screening.

To map the regions responsible for the improved immunogenicity of ST-008, we constructed four pairs of deletion constructs using either JRCSF or ST-008 sequences as a backbone: gp120ΔV3, gp120ΔV1V2V3, gp120 Core, and gp120 Core+V1V2 (Figure 4b). Each of these eight plasmids was used to immunize 8 rabbits with three DNA injections with electroporation followed by one protein boost, this time using the homologous Env glycoprotein. The Day 98 sera obtained two weeks after the protein boost were tested against four pseudoviruses: 6353, BaL, NL4-3, and SF162. The responses induced by the ST-008 gp120ΔV3 were reduced by 2.8- to 17.8-fold compared to the corresponding gp120 sequence (Tables S3 and S4; also compare Figure 4c to a), suggesting that the V3 region is important for eliciting the neutralization activity seen in the previous experiment. Nonetheless, the neutralization activity induced by the modified ST-008 variants was still significantly greater than that induced by the modified JRCSF immunogens.

The results further showed that all four deletion constructs derived from ST-008 induced statistically stronger neutralization responses, when detectable, than did the four JRCSF counterparts (Figure 4c; Table S3). The neutralization activities elicited by all deletion constructs were reduced in this homologous prime-boost immunization experiment compared to that observed with the full-length ST-008 in the heterologous prime-boost experiment. However, the degree of reduction for the ST-008-based deletion constructs was smaller than for the JRCSF counterparts (Table S4). These results suggest that ST-008 has a backbone of conserved sequences that is generally more potent at inducing neutralizing activity compared to JRCSF.

## Discussion

A high rate of mutation allows HIV-1 to escape from typical type-specific antibody responses [55]–[58]. Although several broadly neutralizing mAbs against the Env have been isolated from infected individuals [59]–[65], the conserved epitopes defined by these mAbs are not adequately immunogenic in the viruses that circulate in infected individuals or in current forms of Env-based vaccine candidates [2], [3]. Since the wild-type Env glycoproteins appear incapable of inducing broadly neutralizing antibodies, novel strategies to modify Env must be pursued. One approach for discovery of Env-based vaccine candidates is to use directed molecular evolution to identify proteins that induce broadly neutralizing antibodies. We characterized hundreds of functional gp120 sequences with novel genetic and antigenic properties that are distinct from those of the wild-type Env parents (Figure 1, Figure 2, Figure 3). Systematic *in vivo* testing of these variants identified novel immunogens with clear-cut incremental improvements in neutralization potency (Figure 3, Figure 4).

Although an *in vivo* screening phase is required for vaccine discovery, it is important to use relatively simple but high-throughput *in vitro* assays to reduce the number of variants selected for immunization. In the work reported here, we identified gp120 variants that bound the b12 mAb as an indication of the presence of an intact, broadly conserved neutralizing epitope that overlaps the functional CD4BS of the Env glycoprotein. We also sought b12-positive variants that no longer bound the non-neutralizing b3 and b6 mAbs, which also recognize the CD4BS. The b3 and b6 mAbs are representative of the non-neutralizing antibodies to the CD4BS induced by HIV-1, and we hypothesized that modification of these epitopes might be beneficial in directing the immune response to the conserved b12 neutralizing site. We chose rabbits for *in vivo* screening because the existing broadly neutralizing human mAbs often contain heavy-chain CDR3 loops equal to or longer than 18 amino acids [63], [66], [67]. Wu *et al.* [68] have shown that CDR3 sequences of this length are very rare in mice (0.2%), but much more frequent in rabbits (8.6%) and humans (15%), suggesting a potential advantage of using rabbits over mice for immunization studies.

After *in vitro* DNA recombination of the parental genes, gp120 protein variants with novel antigenic phenotypes were identified at high frequency. The phenotypes represented by categories A, B, C, and F include about 60% of the total variants characterized in an unbiased screen of two libraries (Table 1; Figure 1). It seemed likely that the different antigenic categories would be related to the ability of the gp120 variants to induce neutralizing antibodies in rabbits. However, the variants appear less likely to generate positive neutralizing responses to the sensitive SF162 strain of HIV-1 (Table 1; Figure 3c). Thus, although the ability of a protein to bind an antibody (antigenicity) does not predict its ability to induce the corresponding antibody (immunogenicity), the presence of conserved neutralizing epitopes (defined by binding of 2G12 and b12) in the five improved gp120 immunogens should preserve the chances that effective neutralizing antibodies can be made to them (Figure 3d,e). The ST-008 variant, which induced the most potent neutralizing antibodies, bound b6 and b12 as strongly as did wild-type JRCSF but exhibited reduced binding to b3. This might indicate structural changes in the protein that contribute to its improved neutralization response.

A focus on the lack of binding of non-neutralizing antibodies like b3 or b6 [59] or on the binding of broadly neutralizing mAbs such as 2G12, b12, PG9/16, or VRC01 [59], [61], [65], [69] is perhaps of limited value. The failure to bind a specific antibody such as b6 can result from an inappropriate combination of amino acids that is not necessarily accompanied by the absence of a functional site (e.g., the CD4BS). The broadly neutralizing antibodies are the result of a considerable number of somatic mutation events [69], [70] and optimization of gp120 for strong binding to a specific antibody will create a protein that binds well to the paratope of the mature antibody. However, such a protein will not necessarily bind to the surface-bound germline immunoglobulin of a B cell, binding that is needed to initiate the humoral immune response [71]. Future studies will focus on the use of directed molecular evolution to create immunogens that bind to the germline configuration of broadly neutralizing antibodies.

Env spikes on the viral surface consist of noncovalently associated trimers of gp120/gp41 heterodimers [29], [72]–[74]. Several groups have created soluble trimeric (or oligomeric) forms of Env through various modifications of gp140 [21]–[25], [30], [31], [39] and have reported that they induce more potent neutralizing antibodies than their gp120 counterparts [21], [30], [31], [39]. However, it is not clear whether these marginal improvements were due to the oligomerization or simply due to the inclusion of the T-helper epitopes that are present in the gp41 ectodomain [75], [76]. In a recent study, we found that trimerization *per se* resulted in no improvement in immunogenicity for gp140 trimers [28]. The most potent trimeric gp140 immunogen, gp140-ATC, was no better than the ST-008 protein when immunization was performed with the same protocol (data not shown). Beddows *et al.* [39] studied the immunogenicity of cleaved and stabilized JRFL gp140 SOS-IP trimers and evaluated four of the most potent sera using the same neutralization assay as used here. We therefore compared their Week 20 and Week 54 data (Table 3 in ref. [39]) with our Week 14 (Day 98) data from the eight rabbits immunized with the ST-008 (Figure 4a). As shown in Table S5, the neutralization activity obtained on Week 14 in our study was significantly greater than that obtained on Week 20 in the Beddows *et al.* study. These results demonstrate that soluble Env trimer molecules are not necessarily better immunogens than gp120.

Further work on Env trimers is nonetheless warranted and Kang *et al.* [30] have successfully prepared cleaved and stabilized Env trimers. It is thought that a true mimic of the viral trimeric complex could induce antibodies to conserved quaternary structures of the trimer that would be effective in neutralization of the virus. The PG9/16 and 2909 mAbs bind to a quaternary structure-specific neutralizing epitope and would appear to fulfill this requirement [77]. Env trimers in soluble form or presented on virus-like particles might therefore be able to induce such neutralizing activities.

Our results show that improved immunogens based on the HIV-1 gp120 Env glycoprotein can be identified by application of directed molecular evolution. The more potent neutralizing responses given by ST-008 compared to JRCSF appear to involve multiple factors including a superior response when delivered by DNA vaccination, enhanced V3-based responses, and significantly improved responses to sequences in the relatively conserved regions of gp120. It is unclear what antibody specificities are elicited by ST-008. ST-008 does bind the b6 non-neutralizing antibody, and it is possible that it induces CD4BS antibodies that can neutralize b6-sensitive viruses. However, the ST-008 sera do show neutralization activity against several viruses (92HT594, 92BR020, JRCSF, QH0692, and VLGCG4) that are not sensitive to b6 [54], and this antibody specificity is therefore unlikely to account for the improved neutralizing responses of ST-008 shown in Figure 4a. Deletion of the ST-008 V3 sequences leads to reduced neutralizing responses. This result suggests ST-008 elicits V3-targeting antibodies that contribute to its improved immune response or that loss of V3 in ST-008 alters the conformation of the immunogenic epitope. Deletion of the V3 sequence is not expected to result in large changes to the remainder of gp120 [78], but it is possible that conformational changes outside of V3 contribute to this result. A comparison of the heterologous prime-boost immunization with full-length ST-008 to that of the homologous prime-boost immunizations with deletion constructs further suggests that the full-length Env has qualitatively different immunogenic characteristics than any of the deletion constructs, or that the heterologous boost explains the qualitatively different response. We suggest that both of these factors may be involved, and additional studies will be needed to understand the precise molecular basis of the improved responses elicited by the ST-008. These improvements could not have been readily predicted by the current body of knowledge on Env structure and immunogenicity, and this consideration highlights the unique advantages of the directed molecular evolution approach to immunogen discovery and improvement.

We have focused on gp120 for these initial proof-of-concept studies. However, the approach used here can be applied to other Env-based vaccines including those based on soluble trimers, membrane-bound Env complexes on virus-like particles, or expression by recombinant viral vectors. Further enhancements in immunogenicity can potentially be achieved by additional cycles of the *in vitro* DNA recombination and *in vivo* screening processes, using the improved variants from one round of evolution as the parents in a subsequent round. This recursive aspect of directed molecular evolution is analogous to the principles of natural breeding and classical genetics. The use of directed molecular evolution thus provides a logical, efficient, and powerful strategy for the identification of Env variants with improved immunogenicity.

## Methods

### Cell culture and transient transfection

Chinese Hamster Ovary (CHO)-K1 cells (ATCC No. CRL-61) were maintained at 37°C in serum-containing medium as previous described [79]. All transient transfections of CHO-K1 were performed using LipofectAMINE 2000 (Invitrogen) according to the manufacturer's instructions. At 24 hours post-transfection, cells were washed once, covered with serum-free Opti-MEM I medium (Invitrogen), and incubated for 3 additional days.

### Antibodies, antiserum, and HIV+ human plasma

The human mAbs b3, b6, and b12 recognize discontinuous epitopes that overlap the functionally conserved CD4BS [59], [60]. The 2G12 mAb recognizes a mannose-dependent epitope [61], [62] and was obtained from POLYMUN Scientific, Vienna, Austria. In this study, the IgG1 form of b12 and the Fab form of b3 and b6 were used in some experiments; in the surface plasmon resonance analysis and neutralization assays, the IgG1 forms of both b6 and b12 were used. The HIV^+^ human plasma, N16, obtained from NABI Biopharmaceuticals, exhibited potent and cross-reactive neutralization as described elsewhere [54]. A mouse polyclonal antiserum raised against HIV IIIB gp120 was described previously [28], [79]. Mouse anti-His-tag mAb was from GE Healthcare.

### Expression vector, genes, and sequence analysis

The mammalian expression vector pMAmp [79] was used to clone all *env* genes in this study. Env proteins expressed by pMAmp contain a human tissue plasminogen activator signal peptide as well as an N-terminal hexahistidine tag to facilitate quantitation of expression and protein purification. The wild-type *env* genes encoding full-length gp120 were obtained from ten HIV-1 subtype B strains (Table S1). Selected gp120 genes, including the parental JRCSF and the recombined variants ST-008, ST-040 and ST-080, were human-codon optimized and synthesized based on highly expressed human genes [79], [80].

Four gp120 deletion constructs, gp120ΔV3, gp120ΔV1V2V3, gp120 Core, and gp120 Core+V1V2, were created for both JRCSF and ST-008 from the corresponding humanized genes. The deletion positions for C1, C5, V1/V2, and V3, as well as the replacement Gly-Ala-Gly tripeptide in V1/V2 and V3 deletions, were identical to those of an HXBc2 gp120 Core described previously [81], [82] (see also Figure 4b). Additional gp120 Core constructs were also generated from their wild-type gp120 genes and used as DNA transfection controls (see below).

DNA sequencing was performed using ABI BigDye 3.1 chemistry on an ABI 3700 DNA analyzer. Sequence alignment and similarity for both nucleotide and amino acid sequences were analyzed in Vector NTI 9.0 (Informax). The chimerism of recombined gp120 variants was analyzed using proprietary Maxygen software. Phylogenetic trees were constructed in MEGA 3.0 [83] using the neighbor-joining method and tested by bootstrap analysis using 1,000 replications.

### *In vitro* DNA recombination and construction of gp120 libraries

*In vitro* DNA recombination was carried out essentially as described by Stemmer [84]. The parental *env* gp120 genes are amplified by PCR and randomly fragmented to 50 and 200 base pairs. Various dilutions of the fragments are submitted to a PCR-like thermal cycling reaction to allow them to reassemble by successive elongation reactions [84], [85]. Once gel analysis indicates an accumulation of products in the range of the expected size, the genes are recovered by PCR using two primers that are positioned inside the original PCR primers that were used to prepare the fragments. The PCR products of this reaction are then digested, gel purified, and ligated into the pMAmp vector.

The ligation mixtures were used to transform *Escherichia coli* XL10-Gold® ultracompetent cells (Stratagene) and plated onto Q Trays (Genetix) for an overnight incubation. Individual colonies were identified and picked with the aid of a Q-Bot robot (Genetix) and then seeded into the first ten columns of 96-well plates containing 0.1 ml LB broth with carbenicillin (50 µg/ml). Columns 11 and 12 of 96-well plates were left empty for controls to be added in the subsequent screening steps. After overnight incubation at 37°C, glycerol stocks of the library transformants were prepared with an aid of a Multimek 96/384-Channel Automated Pipettor (Beckman Coulter) and stored at −80°C. The remaining cultures were used to inoculate 96-deep-well blocks containing 1.2 ml of LB with carbenicillin (50 µg/ml) for plasmid preparation. After a 20 hour incubation at 37°C, library plasmids were purified using Qiaprep 96 Turbo Miniprep Kits (Qiagen), quantitated using 96-well UV plates (Costar) by a SpectraMax 190 microplate spectrophotometer (Molecular Devices), normalized to 100 ng/µl using a Tecan Genesis RSP 100 liquid handling system, and stored at −20°C until needed for *in vitro* screening.

### High-throughput *in vitro* screening for antigenicity

CHO-K1 cells were seeded into columns 1 to 11 of 96-well plates at 40,000 cells/well in 100 µl of serum-containing medium and incubated at 37°C for 24 hours. Two µl (100 ng) of each clone from each plate of library plasmids were used to transfect wells in columns 1 to 10 of a corresponding CHO-K1 plate. The same amount of 8 plasmid controls, including the pMAmp vector and the pMAmp constructs expressing JRCSF gp120, 93US073 gp120, 92US727 gp120, 89.6 gp120, JRCSF gp120 Core, 93US073 gp120 Core and 89.6 gp120 Core, respectively, was used to transfect wells 1 through 8 in column 11 of each CHO-K1 plate. Twenty-four hours after transfection, the cells were washed and covered with 200 µl per well of serum-free Opti-MEM I medium and incubated for 3 days. Once harvested, 40 µl of each culture supernatant were diluted into 160 µl of PBS in columns 1–11 of each replicate 96-well plate using the Tecan Genesis RSP 100. To column 12 of each assay plate, 200 µl of 8 1∶2 dilutions of purified JRCSF gp120 protein, starting from 0.5 ng/µl (100 ng per well), were added as a binding signal control. The entire contents of each assay plate were applied to an Optitran BA-S83 membrane (0.2 µm; Schleicher & Schuell) held by a 96-well Minifold I Dot-Blot System (Schleicher & Schuell) with the aid of the Tecan Genesis RSP 100. The membranes were incubated with Blocking Buffer (5% non-fat milk, 1×PBS, 0.05% Tween) and probed with human mAbs diluted in the Blocking Buffer, 250 ng/ml for b3, b6, and b12 and 125 ng/ml for 2G12. The binding signals were visualized by incubation with a secondary antibody conjugated with horseradish peroxidase followed by ECL Plus™ reagents (GE Healthcare). Each blot was exposed to Kodak XAR films multiple times to achieve signals within the linear range of the film for subsequent quantitation using Phoretix Array v2.00 software (Nonlinear Dynamics Ltd). For inter-plate and inter-day comparison, the signal given by each recombined variant was normalized to that of the internal JRCSF gp120 transfection control (column 11, well 2) in the same blot, which was set to 100%. Although 40 µl of culture supernatant were typically used for dot-blot analysis, a clear differentiation between different antibody-binding activities was obtained between 2 to 50 µl of culture supernatant (see Figure 2b and related discussion in Results).

### Immunoprecipitation

CHO-K1 cells were seeded in 6-well plates at 6×10^5^ cells per well in 2 ml of serum-containing medium and grown for 20 hours. Plasmids expressing gp120 were transfected into each well. Twenty-four hours after transfection, the cells were washed once and covered with 3 ml of labeling medium containing 90% DMEM without Met, 10% dialyzed fetal bovine serum, 1% of 100× stocks of L-glutamine, sodium pyruvate, and penicillin-streptomycin, respectively (all from Invitrogen) and incubated for 1 hour. Medium was removed and replaced with 1 ml of labeling medium+200 mCi ^35^S-Met/Cys (GE Healthcare). Culture supernatants were harvested 24 hours later and stored at −20°C. For each immunoprecipitation reaction, 30 µl of well-suspended Protein L-agarose (Genomics One International) were washed with 300 µl of PBS, resuspended into 300 µl of PBS along with either 4 µg of human mAb or 5 µl of the mouse polyclonal antiserum, and incubated with slow rotation at 4°C for 1 hour to allow the formation of an antibody-Protein L-agarose complex. The complex was washed three times with 300 µl of the serum medium, resuspended into 300 µl of the same medium, and mixed with 300 µl of ^35^S-label culture supernatant. The reaction was incubated at 4°C for 2 hours with slow rotation, followed by three 10-minute washes with 500 mM NaCl, 0.02% sodium azide, 0.1% Triton ×100 in PBS, and one final wash with PBS. After the final spin, the gp120-antibody-Protein L-agarose complex was resuspended into 15 µl of 1× LDS loading buffer (Invitrogen) and separated on a 4–12% SDS-PAGE gel (Invitrogen). The gel was dried and exposed to a low-energy storage phosphor screen (GE Healthcare) at room temperature overnight. The image was acquired and quantitated using a Storm PhosphorImager with ImageQuant System (GE Healthcare).

### Stable cell lines and protein purification

Stable CHO cell lines expressing JRCSF gp120, ST-040, ST-080, and four ST-008 gp120 deletion constructs (Figure 4b) were generated using a procedure described previously [79]. Both production and the metal-chelating affinity purification of these proteins were also previously reported [79]. In the course of purifying different gp120 proteins, we have observed that one-step affinity chromatography does not always produce protein of sufficient purity. In those cases, we developed a cleanup procedure using a High Q anion exchange column (Bio-Rad) and a hydroxyapatite CHT II column (Bio-Rad) to remove impurities [79]. Using a combination of affinity chromatography and cleanup procedure, we were able to achieve a desirable purity (>95%) for both kinetic analysis and animal immunization.

### Surface plasmon resonance analysis

A CM5 chip (Biacore) was used to immobilized 18,000 response units (RU) of goat anti-human gamma chain antibody (KPL, Inc.) for each of the four flow paths by flowing 50 µg/ml of the antibody in pH 5.0 acetate buffer (Biacore) at a rate of 10 µl per minute on a Biacore 2000 (GE Healthcare). The human IgG1 mAbs b6 and b12 (100 RU per cycle) were captured on the surfaces of flow paths 2 and 4 at a flow rate of 10 µl per minute. Flow paths 1 and 3 remained unbound to serve as a reference surface. A range of concentrations of purified gp120 (0, 0.78, 1.56, 3.13, 6.25, 12.5, 25, 50, 100 and 200 nM) was injected onto the antibody surface in random order, with 25 nM repeated in one additional cycle, at a flow rate of 30 µl per minute for 2 minutes, followed by a 5 minute dissociation time. After each association and dissociation cycle, the goat anti-human gamma chain surface was regenerated by a 1-minute run followed by a 30-second run of 20 mM HCl at a flow rate of 10 µl per minute. Then, 100 RU of fresh b6 and b12 were recaptured on the surfaces of flow paths 2 and 4, respectively, for the next cycle. Sensor data were prepared for kinetic analysis by subtracting the binding response collected from the corresponding goat anti-human gamma chain reference surface. The association and dissociation data were fitted simultaneously to a single-site binding with local mass transfer model by using BIAevaluation software (Biacore).

### Rabbit immunizations

Selected plasmid constructs expressing gp120 variants were used for mg-scale endotoxin-free DNA preparation and subsequent animal immunization at Aldevron LLC (Fargo, ND) under animal use protocol 03001-2011-08-2 Rabbit Electroporation approved by the local Animal Care and Use Committee. Certified parasite-free female New Zealand White rabbits were obtained at 8 weeks of age with an average weight of 1.8–2.3 kg. After a one-week acclimation period, each rabbit was immunized intramuscularly in the *triceps brachii* with three injections of DNA on Days 0, 28 and 56. A total 400 µg of DNA per injection (200 µg in each hind limb) was delivered with electroporation. On Day 84, each rabbit received an intraperitoneal injection of 100 µg of JRCSF gp120 protein mixed with 20 µl of aluminum hydroxide gel adjuvant (ALHYDROGEL “85” 2%, Brenntag Biostor) and brought to a final volume of 1 ml with endotoxin-free PBS (Sigma). Test bleeds were obtained on Day 0 and 2 weeks after the third DNA injection on Day 70. Terminal bleeds were collected 2 weeks after protein boosting on Day 98.

In the study of four gp120 deletion pairs of JRCSF and ST-008 (Figure 4c), each rabbit received an intramuscular injection of 100 µg of homologous protein mixed with AS02A (GlaxoSmithKline Biologicals) on Day 84 as described elsewhere [28]. Although AS02A-formulated protein vaccine candidates has shown efficacy in both human clinical testing [86] and a monkey challenge model [87], we found that there is no significant difference between AS02A and aluminum gel when immunizing rabbits with the 3×DNA electroporation plus 1×protein boost protocol (data not shown), except that AS02A is much easier to handle than Alum.

### Neutralization assays

The neutralizing activities of mAbs, purified antibodies, or rabbit sera were determined using a pseudovirus-based system as previously described [28], [55], [57]. In the initial *in vivo* screening of library variants in which 2 rabbits per clone were utilized, we measured the percent neutralization at a single dilution point using antibodies purified from rabbit sera using Montage Antibody Purification Kits (Millipore) to minimize any background interference (Figure 3c,d). In confirmation studies, in which 8 rabbits were used for each construct, rabbit serum was use to determine the IC50 neutralization titers (Figure 4a, c).

### Statistical analysis

Two-tailed homoscedastic t-tests were performed by using a log10 transform of the IC50 values to compare sera from the JRCSF and the ST-008 groups (Figure 4a, c). *P* values less than 0.05 were considered statistically significant.

### Accession codes

DNA sequences encoding gp120 variants are deposited in GenBank with the following accession codes: ST-003 (HI550410), ST-008 (HI550369), ST-051 (HI550370), ST-057 (HI550411), ST-080 (HI550371), ST-128 (HI550412), ST-140 (HI550372), ST-148 (HI550373), ST-161 (HI550374), ST-168 (HI550413), ST-173 (HI550414), ST-188 (HI550375), ST-194 (HI550415), ST-199 (HI550416), and ST-272 (HI550417).

## Supporting Information

Figure S1 Amino acid sequences of JRCSF and ST-008 gp120 proteins. Sequence alignment by ClustalW of JRCSF gp120 (GenBank: AAB03749) and ST-008 excluding the tPA leader sequence and spacer. Identities = 439/488 (89%), Positives = 457/488 (93%), Gaps = 12/488 (2%).
(TIFF)

Table S1 Subtype B HIV-1 *env* genes used for *in vitro* DNA recombination.
(DOC)

Table S2 Comparison of kinetic constants for the interactions between mAbs and wild-type and recombined gp120 proteins.
(DOC)

Table S3 Comparison of Day 98 sera induced by gp120 deletion constructs.
(DOC)

Table S4 Reduction of neutralization potency in Day 98 sera induced by gp120 deletion constructs.
(DOC)

Table S5 Comparison of potency of two Env immunogens after protein boost.
(DOC)

## References

[1] Stamatatos L, Morris L, Burton DR, Mascola JR (2009). Neutralizing antibodies generated during natural HIV-1 infection: good news for an HIV-1 vaccine?. Nat Med.

[2] Walker BD, Burton DR (2008). Toward an AIDS vaccine.. Science.

[3] Karlsson Hedestam GB, Fouchier RA, Phogat S, Burton DR, Sodroski J (2008). The challenges of eliciting neutralizing antibodies to HIV-1 and to influenza virus.. Nat Rev Microbiol.

[4] Haynes BF, Montefiori DC (2006). Aiming to induce broadly reactive neutralizing antibody responses with HIV-1 vaccine candidates.. Expert Rev Vaccines.

[5] Srivastava IK, Ulmer JB, Barnett SW (2005). Role of neutralizing antibodies in protective immunity against HIV.. Hum Vaccin.

[6] Binley JM, Lybarger EA, Crooks ET, Seaman MS, Gray E (2008). Profiling the specificity of neutralizing antibodies in a large panel of plasmas from patients chronically infected with human immunodeficiency virus type 1 subtypes B and C.. J Virol.

[7] Sather DN, Armann J, Ching LK, Mavrantoni A, Sellhorn G (2009). Factors associated with the development of cross-reactive neutralizing antibodies during human immunodeficiency virus type 1 infection.. J Virol.

[8] Carotenuto P, Looij D, Keldermans L, de Wolf F, Goudsmit J (1998). Neutralizing antibodies are positively associated with CD4+ T-cell counts and T-cell function in long-term AIDS-free infection.. Aids.

[9] Li Y, Migueles SA, Welcher B, Svehla K, Phogat A (2007). Broad HIV-1 neutralization mediated by CD4-binding site antibodies.. Nat Med.

[10] Pilgrim AK, Pantaleo G, Cohen OJ, Fink LM, Zhou JY (1997). Neutralizing antibody responses to human immunodeficiency virus type 1 in primary infection and long-term-nonprogressive infection.. J Infect Dis.

[11] Hessell AJ, Poignard P, Hunter M, Hangartner L, Tehrani DM (2009). Effective, low-titer antibody protection against low-dose repeated mucosal SHIV challenge in macaques.. Nat Med.

[12] Nishimura Y, Igarashi T, Haigwood N, Sadjadpour R, Plishka RJ (2002). Determination of a statistically valid neutralization titer in plasma that confers protection against simian-human immunodeficiency virus challenge following passive transfer of high-titered neutralizing antibodies.. J Virol.

[13] Shibata R, Igarashi T, Haigwood N, Buckler-White A, Ogert R (1999). Neutralizing antibody directed against the HIV-1 envelope glycoprotein can completely block HIV-1/SIV chimeric virus infections of macaque monkeys.. Nat Med.

[14] Baba TW, Liska V, Hofmann-Lehmann R, Vlasak J, Xu W (2000). Human neutralizing monoclonal antibodies of the IgG1 subtype protect against mucosal simian-human immunodeficiency virus infection.. Nat Med.

[15] Hofmann-Lehmann R, Vlasak J, Rasmussen RA, Smith BA, Baba TW (2001). Postnatal passive immunization of neonatal macaques with a triple combination of human monoclonal antibodies against oral simian-human immunodeficiency virus challenge.. J Virol.

[16] Mascola JR (2003). Defining the protective antibody response for HIV-1.. Curr Mol Med.

[17] (2003). AIDSVAX fails to prove efficacious in large-scale trial.. Expert Rev Anti Infect Ther.

[18] Rerks-Ngarm S, Pitisuttithum P, Nitayaphan S, Kaewkungwal J, Chiu J (2009). Vaccination with ALVAC and AIDSVAX to prevent HIV-1 infection in Thailand.. N Engl J Med.

[19] Kwong PD, Wilson IA (2009). HIV-1 and influenza antibodies: seeing antigens in new ways.. Nat Immunol.

[20] Li Y, Cleveland B, Klots I, Travis B, Richardson BA (2008). Removal of a single N-linked glycan in human immunodeficiency virus type 1 gp120 results in an enhanced ability to induce neutralizing antibody responses.. J Virol.

[21] Zhang PF, Cham F, Dong M, Choudhary A, Bouma P (2007). Extensively cross-reactive anti-HIV-1 neutralizing antibodies induced by gp140 immunization.. Proc Natl Acad Sci U S A.

[22] Binley JM, Sanders RW, Clas B, Schuelke N, Master A (2000). A recombinant human immunodeficiency virus type 1 envelope glycoprotein complex stabilized by an intermolecular disulfide bond between the gp120 and gp41 subunits is an antigenic mimic of the trimeric virion-associated structure.. J Virol.

[23] Selvarajah S, Puffer BA, Lee FH, Zhu P, Li Y (2008). Focused dampening of antibody response to the immunodominant variable loops by engineered soluble gp140.. AIDS Res Hum Retroviruses.

[24] Yang X, Lee J, Mahony EM, Kwong PD, Wyatt R (2002). Highly stable trimers formed by human immunodeficiency virus type 1 envelope glycoproteins fused with the trimeric motif of T4 bacteriophage fibritin.. J Virol.

[25] Yang X, Farzan M, Wyatt R, Sodroski J (2000). Characterization of stable, soluble trimers containing complete ectodomains of human immunodeficiency virus type 1 envelope glycoproteins.. J Virol.

[26] Morner A, Douagi I, Forsell MN, Sundling C, Dosenovic P (2009). Human immunodeficiency virus type 1 env trimer immunization of macaques and impact of priming with viral vector or stabilized core protein.. J Virol.

[27] Hammonds J, Chen X, Fouts T, DeVico A, Montefiori D (2005). Induction of neutralizing antibodies against human immunodeficiency virus type 1 primary isolates by Gag-Env pseudovirion immunization.. J Virol.

[28] Du SX, Idiart RJ, Mariano EB, Chen H, Jiang P (2009). Effect of trimerization motifs on quaternary structure, antigenicity, and immunogenicity of a noncleavable HIV-1 gp140 envelope glycoprotein.. Virology.

[29] Crooks ET, Moore PL, Franti M, Cayanan CS, Zhu P (2007). A comparative immunogenicity study of HIV-1 virus-like particles bearing various forms of envelope proteins, particles bearing no envelope and soluble monomeric gp120.. Virology.

[30] Kang YK, Andjelic S, Binley JM, Crooks ET, Franti M (2009). Structural and immunogenicity studies of a cleaved, stabilized envelope trimer derived from subtype A HIV-1.. Vaccine.

[31] Yang X, Wyatt R, Sodroski J (2001). Improved elicitation of neutralizing antibodies against primary human immunodeficiency viruses by soluble stabilized envelope glycoprotein trimers.. J Virol.

[32] Dey B, Svehla K, Xu L, Wycuff D, Zhou T (2009). Structure-based stabilization of HIV-1 gp120 enhances humoral immune responses to the induced co-receptor binding site.. PLoS Pathog.

[33] Wang S, Kennedy JS, West K, Montefiori DC, Coley S (2008). Cross-subtype antibody and cellular immune responses induced by a polyvalent DNA prime-protein boost HIV-1 vaccine in healthy human volunteers.. Vaccine.

[34] Wang S, Pal R, Mascola JR, Chou TH, Mboudjeka I (2006). Polyvalent HIV-1 Env vaccine formulations delivered by the DNA priming plus protein boosting approach are effective in generating neutralizing antibodies against primary human immunodeficiency virus type 1 isolates from subtypes A, B, C, D and E.. Virology.

[35] Derby NR, Kraft Z, Kan E, Crooks ET, Barnett SW (2006). Antibody responses elicited in macaques immunized with human immunodeficiency virus type 1 (HIV-1) SF162-derived gp140 envelope immunogens: comparison with those elicited during homologous simian/human immunodeficiency virus SHIVSF162P4 and heterologous HIV-1 infection.. J Virol.

[36] Barnett SW, Lu S, Srivastava I, Cherpelis S, Gettie A (2001). The ability of an oligomeric human immunodeficiency virus type 1 (HIV-1) envelope antigen to elicit neutralizing antibodies against primary HIV-1 isolates is improved following partial deletion of the second hypervariable region.. J Virol.

[37] Pantophlet R, Wilson IA, Burton DR (2004). Improved design of an antigen with enhanced specificity for the broadly HIV-neutralizing antibody b12.. Protein Eng Des Sel.

[38] Pantophlet R, Wilson IA, Burton DR (2003). Hyperglycosylated mutants of human immunodeficiency virus (HIV) type 1 monomeric gp120 as novel antigens for HIV vaccine design.. J Virol.

[39] Beddows S, Schulke N, Kirschner M, Barnes K, Franti M (2005). Evaluating the immunogenicity of a disulfide-stabilized, cleaved, trimeric form of the envelope glycoprotein complex of human immunodeficiency virus type 1.. J Virol.

[40] Doria-Rose NA, Learn GH, Rodrigo AG, Nickle DC, Li F (2005). Human immunodeficiency virus type 1 subtype B ancestral envelope protein is functional and elicits neutralizing antibodies in rabbits similar to those elicited by a circulating subtype B envelope.. J Virol.

[41] Kothe DL, Decker JM, Li Y, Weng Z, Bibollet-Ruche F (2007). Antigenicity and immunogenicity of HIV-1 consensus subtype B envelope glycoproteins.. Virology.

[42] Kothe DL, Li Y, Decker JM, Bibollet-Ruche F, Zammit KP (2006). Ancestral and consensus envelope immunogens for HIV-1 subtype C.. Virology.

[43] Liao HX, Sutherland LL, Xia SM, Brock ME, Scearce RM (2006). A group M consensus envelope glycoprotein induces antibodies that neutralize subsets of subtype B and C HIV-1 primary viruses.. Virology.

[44] Crameri A, Raillard SA, Bermudez E, Stemmer WP (1998). DNA shuffling of a family of genes from diverse species accelerates directed evolution.. Nature.

[45] Ness JE, Kim S, Gottman A, Pak R, Krebber A (2002). Synthetic shuffling expands functional protein diversity by allowing amino acids to recombine independently.. Nat Biotechnol.

[46] Castle LA, Siehl DL, Gorton R, Patten PA, Chen YH (2004). Discovery and directed evolution of a glyphosate tolerance gene.. Science.

[47] Lazetic S, Leong SR, Chang JC, Ong R, Dawes G (2002). Chimeric costimulatory molecules that selectively act through CD28 or CTLA-4 on human T cells.. J Biol Chem.

[48] Leong SR, Chang JC, Ong R, Dawes G, Stemmer WP (2003). Optimized expression and specific activity of IL-12 by directed molecular evolution.. Proc Natl Acad Sci U S A.

[49] Brideau-Andersen AD, Huang X, Sun SC, Chen TT, Stark D (2007). Directed evolution of gene-shuffled IFN-alpha molecules with activity profiles tailored for treatment of chronic viral diseases.. Proc Natl Acad Sci U S A.

[50] Bacher JM, Reiss BD, Ellington AD (2002). Anticipatory evolution and DNA shuffling.. Genome Biol.

[51] Kurtzman AL, Govindarajan S, Vahle K, Jones JT, Heinrichs V (2001). Advances in directed protein evolution by recursive genetic recombination: applications to therapeutic proteins.. Curr Opin Biotechnol.

[52] Apt D, Raviprakash K, Brinkman A, Semyonov A, Yang S (2006). Tetravalent neutralizing antibody response against four dengue serotypes by a single chimeric dengue envelope antigen.. Vaccine.

[53] Dupuy LC, Locher CP, Paidhungat M, Richards MJ, Lind CM (2009). Directed molecular evolution improves the immunogenicity and protective efficacy of a Venezuelan equine encephalitis virus DNA vaccine.. Vaccine.

[54] Binley JM, Wrin T, Korber B, Zwick MB, Wang M (2004). Comprehensive cross-clade neutralization analysis of a panel of anti-human immunodeficiency virus type 1 monoclonal antibodies.. J Virol.

[55] Richman DD, Wrin T, Little SJ, Petropoulos CJ (2003). Rapid evolution of the neutralizing antibody response to HIV type 1 infection.. Proc Natl Acad Sci U S A.

[56] Frost SD, Wrin T, Smith DM, Kosakovsky Pond SL, Liu Y (2005). Neutralizing antibody responses drive the evolution of human immunodeficiency virus type 1 envelope during recent HIV infection.. Proc Natl Acad Sci U S A.

[57] Frost SD, Liu Y, Pond SL, Chappey C, Wrin T (2005). Characterization of human immunodeficiency virus type 1 (HIV-1) envelope variation and neutralizing antibody responses during transmission of HIV-1 subtype B.. J Virol.

[58] Wei X, Decker JM, Wang S, Hui H, Kappes JC (2003). Antibody neutralization and escape by HIV-1.. Nature.

[59] Roben P, Moore JP, Thali M, Sodroski J, Barbas CF (1994). Recognition properties of a panel of human recombinant Fab fragments to the CD4 binding site of gp120 that show differing abilities to neutralize human immunodeficiency virus type 1.. J Virol.

[60] Pantophlet R, Ollmann Saphire E, Poignard P, Parren PW, Wilson IA (2003). Fine mapping of the interaction of neutralizing and nonneutralizing monoclonal antibodies with the CD4 binding site of human immunodeficiency virus type 1 gp120.. J Virol.

[61] Trkola A, Purtscher M, Muster T, Ballaun C, Buchacher A (1996). Human monoclonal antibody 2G12 defines a distinctive neutralization epitope on the gp120 glycoprotein of human immunodeficiency virus type 1.. J Virol.

[62] Scanlan CN, Pantophlet R, Wormald MR, Ollmann Saphire E, Stanfield R (2002). The broadly neutralizing anti-human immunodeficiency virus type 1 antibody 2G12 recognizes a cluster of alpha1→2 mannose residues on the outer face of gp120.. J Virol.

[63] Kunert R, Wolbank S, Stiegler G, Weik R, Katinger H (2004). Characterization of molecular features, antigen-binding, and in vitro properties of IgG and IgM variants of 4E10, an anti-HIV type 1 neutralizing monoclonal antibody.. AIDS Res Hum Retroviruses.

[64] Wu X, Yang ZY, Li Y, Hogerkorp CM, Schief WR (2010). Rational design of envelope identifies broadly neutralizing human monoclonal antibodies to HIV-1.. Science.

[65] Walker LM, Phogat SK, Chan-Hui PY, Wagner D, Phung P (2009). Broad and potent neutralizing antibodies from an African donor reveal a new HIV-1 vaccine target.. Science.

[66] Saphire EO, Parren PW, Pantophlet R, Zwick MB, Morris GM (2001). Crystal structure of a neutralizing human IgG against HIV-1: a template for vaccine design.. Science.

[67] Barbas CF, Collet TA, Amberg W, Roben P, Binley JM (1993). Molecular profile of an antibody response to HIV-1 as probed by combinatorial libraries.. J Mol Biol.

[68] Wu TT, Johnson G, Kabat EA (1993). Length distribution of CDRH3 in antibodies.. Proteins.

[69] Zhou T, Georgiev I, Wu X, Yang ZY, Dai K (2010). Structural basis for broad and potent neutralization of HIV-1 by antibody VRC01.. Science.

[70] Pancera M, McLellan JS, Wu X, Zhu J, Changela A (2010). Crystal structure of PG16 and chimeric dissection with somatically related PG9: Structure-function analysis of two quaternary-specific antibodies that effectively neutralize HIV-1.. J Virol.

[71] Xiao X, Chen W, Feng Y, Zhu Z, Prabakaran P (2009). Germline-like predecessors of broadly neutralizing antibodies lack measurable binding to HIV-1 envelope glycoproteins: implications for evasion of immune responses and design of vaccine immunogens.. Biochem Biophys Res Commun.

[72] Liu J, Bartesaghi A, Borgnia MJ, Sapiro G, Subramaniam S (2008). Molecular architecture of native HIV-1 gp120 trimers.. Nature.

[73] Zhu P, Liu J, Bess J, Chertova E, Lifson JD (2006). Distribution and three-dimensional structure of AIDS virus envelope spikes.. Nature.

[74] Zanetti G, Briggs JA, Grunewald K, Sattentau QJ, Fuller SD (2006). Cryo-electron tomographic structure of an immunodeficiency virus envelope complex in situ.. PLoS Pathog.

[75] Grundner C, Pancera M, Kang JM, Koch M, Sodroski J (2004). Factors limiting the immunogenicity of HIV-1 gp120 envelope glycoproteins.. Virology.

[76] Weaver EA, Lu Z, Camacho ZT, Moukdar F, Liao HX (2006). Cross-subtype T-cell immune responses induced by a human immunodeficiency virus type 1 group m consensus env immunogen.. J Virol.

[77] Wu X, Changela A, O'Dell S, Schmidt SD, Pancera M (2011). Immunotypes of a Quaternary Site of HIV-1 Vulnerability and Their Recognition by Antibodies.. Journal of virology.

[78] Huang CC, Tang M, Zhang MY, Majeed S, Montabana E (2005). Structure of a V3-containing HIV-1 gp120 core.. Science.

[79] Du SX, Xu L, Viswanathan S, Whalen RG (2008). Inhibition of V3-specific cleavage of recombinant HIV-1 gp120 produced in Chinese hamster ovary cells.. Protein Expr Purif.

[80] Haas J, Park EC, Seed B (1996). Codon usage limitation in the expression of HIV-1 envelope glycoprotein.. Curr Biol.

[81] Kwong PD, Wyatt R, Robinson J, Sweet RW, Sodroski J (1998). Structure of an HIV gp120 envelope glycoprotein in complex with the CD4 receptor and a neutralizing human antibody.. Nature.

[82] Wyatt R, Kwong PD, Desjardins E, Sweet RW, Robinson J (1998). The antigenic structure of the HIV gp120 envelope glycoprotein.. Nature.

[83] Kumar S, Tamura K, Nei M (2004). MEGA3: Integrated software for Molecular Evolutionary Genetics Analysis and sequence alignment.. Brief Bioinform.

[84] Stemmer WP (1994). DNA shuffling by random fragmentation and reassembly: in vitro recombination for molecular evolution.. Proc Natl Acad Sci U S A.

[85] Minshull J, Govindarajan S, Cox T, Ness JE, Gustafsson C (2004). Engineered protein function by selective amino acid diversification.. Methods.

[86] Bojang K, Milligan P, Pinder M, Doherty T, Leach A (2009). Five-year safety and immunogenicity of GlaxoSmithKline's candidate malaria vaccine RTS,S/AS02 following administration to semi-immune adult men living in a malaria-endemic region of The Gambia.. Hum Vaccin.

[87] Mooij P, van der Kolk M, Bogers WM, ten Haaft PJ, Van Der Meide P (1998). A clinically relevant HIV-1 subunit vaccine protects rhesus macaques from in vivo passaged simian-human immunodeficiency virus infection.. AIDS.

