# Supplementary figures and images for: A Directed Molecular Evolution Approach to Improved Immunogenicity of the HIV-1 Envelope Glycoprotein
Source: PLoS One. 2011 Jun 29;6(6):e20927. doi: 10.1371/journal.pone.0020927 (PMC3126809; doi:10.1371/journal.pone.0020927)

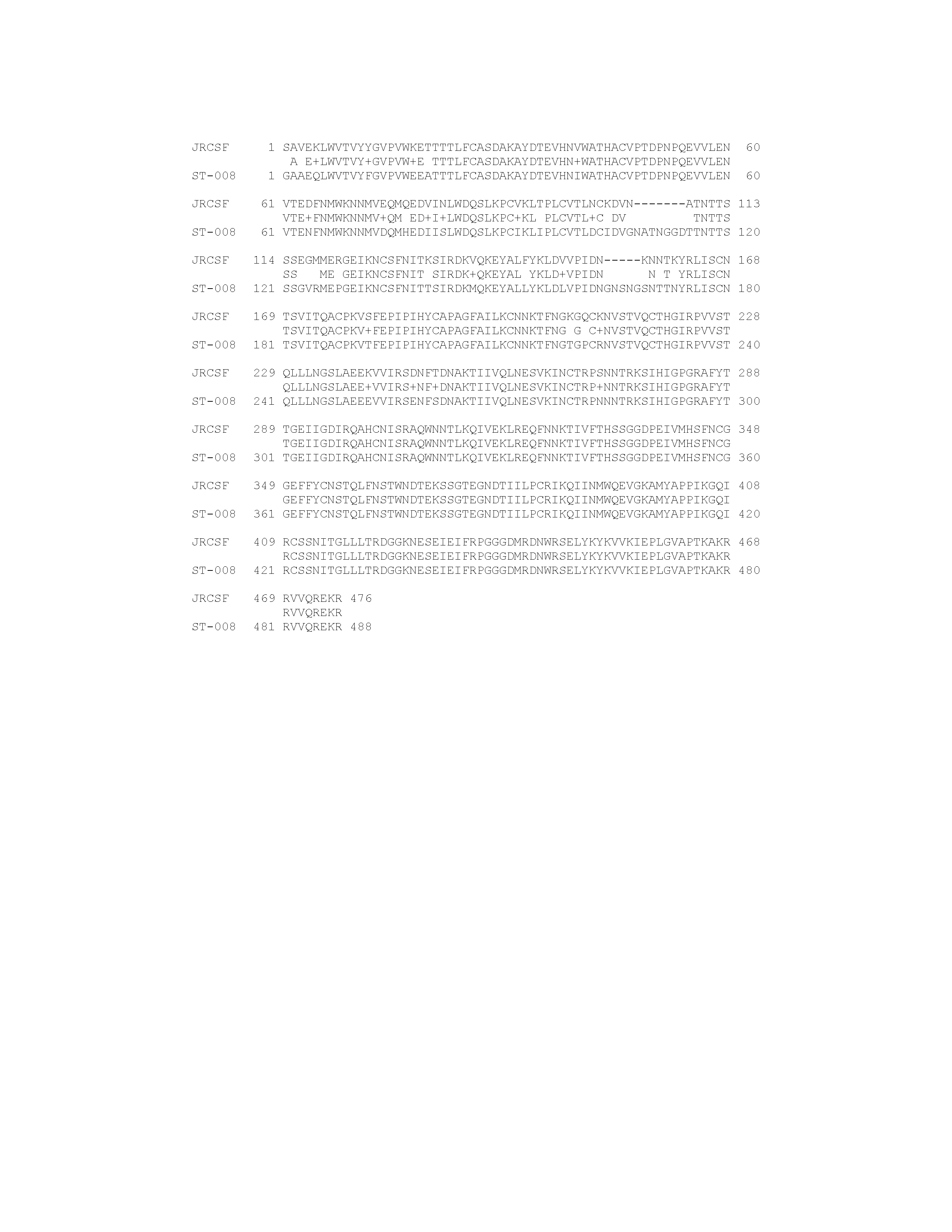

Supplement: Figure S1 — Amino acid sequences of JRCSF and ST-008 gp120 proteins. Sequence alignment by ClustalW of JRCSF gp120 (GenBank: AAB03749) and ST-008 excluding the tPA leader sequence and spacer. Identities = 439/488 (89%), Positives = 457/488 (93%), Gaps = 12/488 (2%). (TIFF) [file pone.0020927.s001.tif]
