# Supplementary material for: A Directed Molecular Evolution Approach to Improved Immunogenicity of the HIV-1 Envelope Glycoprotein
Source: PLoS One. 2011 Jun 29;6(6):e20927. doi: 10.1371/journal.pone.0020927 (PMC3126809; doi:10.1371/journal.pone.0020927)
Supplement: Table S1 — Subtype B HIV-1 env genes used for in vitro DNA recombination. (DOC) [file pone.0020927.s002.doc]

Supplementary Table 1

Subtype B HIV-1 *env* genes used for *in vitro* DNA recombination

| **Viral Strain** | **GenBank Accession No.** | **Phenotype*** | **Co-receptor Usage**** |
| --- | --- | --- | --- |
| JRCSF | AY426125 | NSI | R5 |
| 89.6 | U39362 | SI | R5X4 |
| 92HT593 | AY669721 | SI | R5X4 |
| 92HT594 | U08445 | SI | R5X4 |
| 92HT596 | U08446 | SI | R5X4 |
| 92HT599 | U08447 | SI | X4 |
| 92US657 | U04908 | NSI | R5 |
| 92US712 | AY669725 | NSI | R5 |
| 92US727 | U79720 | NSI | R5 |
| 93US073 | AY669727 | NSI | R5 |

* NSI: non-syncytium inducing; SI: syncytium inducing

** R5, CCR5-tropic; X4, CXCR4-tropic
