# Supplementary material for: A Directed Molecular Evolution Approach to Improved Immunogenicity of the HIV-1 Envelope Glycoprotein
Source: PLoS One. 2011 Jun 29;6(6):e20927. doi: 10.1371/journal.pone.0020927 (PMC3126809; doi:10.1371/journal.pone.0020927)
Supplement: Table S2 — Comparison of kinetic constants for the interactions between mAbs and wild-type and recombined gp120 proteins. (DOC) [file pone.0020927.s003.doc]

Supplementary Table 2

Comparison of kinetic constants for the interactions between mAbs and wild-type and recombined gp120 proteins

| **Protein** | **Binding affinity to b12 IgG** | | | |  | **Binding affinity to b6 IgG** | | | |
| --- | --- | --- | --- | --- | --- | --- | --- | --- | --- |
|  | **ka** | **kd** | **Ka** | **Fold Increase** |  | **ka** | **Kd** | **Ka** | **Fold Decrease** |
| JRCSF | 1.73 x 10^5^ | 4.77 x 10^-3^ | 3.62 x 10^7^ | 1 |  | 1.71 x 10^5^ | 4.20 x 10^-7^* | 4.08 x 10^11^* | 1 |
| ST-080 | 1.80 x 10^5^ | 1.47 x 10^-3^ | 1.23 x 10^8^ | 3.4 |  | 5.55 x 10^4^ | 3.66 x 10^-3^ | 1.52 x 10^7^ | ≥2.7 x 10^4*^ |
| ST-140 | 1.25 x 10^5^ | 1.65 x 10^-3^ | 7.62 x 10^7^ | 2.1 |  | N.D.** | N.D. | N.D. | ≥4.1 x 10^11^* |

* Although these numbers were obtained from the best curve fit, the dissociation rate was too slow to be accurately detected by the Biacore 2000.

** Not detectable
