# Supplementary material for: A Directed Molecular Evolution Approach to Improved Immunogenicity of the HIV-1 Envelope Glycoprotein
Source: PLoS One. 2011 Jun 29;6(6):e20927. doi: 10.1371/journal.pone.0020927 (PMC3126809; doi:10.1371/journal.pone.0020927)
Supplement: Table S3 — Comparison of Day 98 sera induced by gp120 deletion constructs. (DOC) [file pone.0020927.s004.doc]

Supplementary Table 3

Comparison of Day 98 sera induced by gp120 deletion constructs

| gp120 Deletion Constructs | Pseudo viruses | Comparison of D98 Sera (GMT*) | | | |
| --- | --- | --- | --- | --- | --- |
|  |  | JRCSF backbone | ST-008  backbone | Fold increase  (ST-008/JRCSF) | *P* Value** |
| gp120ΔV3 | SF162 | 449 | 926 | 2.1 | 0.008 |
|  | NL4-3 | 95 | 257 | 2.7 | 0.003 |
|  | BaL | 10 | 15 | 1.5 | 0.037 |
|  | 6535 | 10 | 15 | 1.5 | 0.007 |
| gp120ΔV1V2V3 | SF162 | 269 | 1199 | 4.5 | <0.001 |
|  | NL4-3 | 82 | 732 | 8.9 | <0.001 |
|  | BaL | 10 | 19 | 1.9 | 0.002 |
|  | 6535 | 11 | 22 | 2.2 | 0.005 |
| gp120 Core | SF162 | 136 | 629 | 4.6 | 0.009 |
|  | NL4-3 | 34 | 188 | 5.5 | 0.003 |
| gp120 Core+ V1V2 | SF162 | 228 | 810 | 3.6 | 0.009 |
|  | NL4-3 | 40 | 161 | 4.0 | 0.025 |
| * D98 sera from indicate gp120 deletion constructs and backbone were examined against four pseudoviruses. The geometric mean of IC50 neutralization titers (GMT) was calculated for each immunization group.  ** Two-tailed homoscedastic t-test using log_10_ (IC50); *n* = 8 | | | | | |
