# Supplementary material for: A Directed Molecular Evolution Approach to Improved Immunogenicity of the HIV-1 Envelope Glycoprotein
Source: PLoS One. 2011 Jun 29;6(6):e20927. doi: 10.1371/journal.pone.0020927 (PMC3126809; doi:10.1371/journal.pone.0020927)
Supplement: Table S4 — Reduction of neutralization potency in Day 98 sera induced by gp120 deletion constructs. (DOC) [file pone.0020927.s005.doc]

**Supplementary Table 4**

Reduction of neutralization potency in Day 98 sera induced by gp120 deletion constructs

| gp120 Deletion Constructs | Pseudo viruses | Fold reduction of GMT compared to gp120 immunized groups* | | |
| --- | --- | --- | --- | --- |
|  |  | JRCSF  backbone | ST-008 backbone | Difference in  fold of reduction  (JRCSF/ST-008) |
| gp120ΔV3 | SF162 | 10.0 | 6.6 | 1.52 |
|  | NL4-3 | 4.4 | 2.6 | 1.67 |
|  | BaL | 24.8 | 17.8 | 1.39 |
|  | 6535 | 2.8 | 5.3 | 0.53 |
| gp120ΔV1V2V3 | SF162 | 16.7 | 5.1 | 3.29 |
|  | NL4-3 | 5.1 | 0.9 | 5.52 |
|  | BaL | 24.8 | 14.1 | 1.76 |
|  | 6535 | 2.5 | 3.6 | 0.71 |
| gp120 Core | SF162 | 33.0 | 9.6 | 3.42 |
|  | NL4-3 | 12.3 | 3.6 | 3.42 |
| gp120 Core+ V1V2 | SF162 | 19.7 | 7.5 | 2.63 |
|  | NL4-3 | 10.5 | 4.2 | 2.49 |
| The fold reduction was calculated by dividing the GMT of Day 98 sera from the gp120 immunization group (Fig. 4a) by that of the indicated deletion constructs. | | | | |
