# Supplementary material for: A Directed Molecular Evolution Approach to Improved Immunogenicity of the HIV-1 Envelope Glycoprotein
Source: PLoS One. 2011 Jun 29;6(6):e20927. doi: 10.1371/journal.pone.0020927 (PMC3126809; doi:10.1371/journal.pone.0020927)
Supplement: Table S5 — Comparison of potency of two Env immunogens after protein boost. (DOC) [file pone.0020927.s006.doc]

**Supplementary Table 5**

Comparison of potency of two Env immunogens after protein boost

| **Immunogen** | **Studies** | **Time of**  **bleed** | **Immunization events*** | **GMT against Pseudoviruses** | | | | | |
| --- | --- | --- | --- | --- | --- | --- | --- | --- | --- |
|  |  |  |  | **SF162** | **NL4-3** | **BaL** | **1196** | **JR-FL** | **JRCSF** |
| JRFL gp140  SOS-IP trimer | Beddows *et al.* [39] | 20 weeks | 2 X D + 2 X P | 1740 | 131 | 42 | 40 | — | — |
|  |  | 54 weeks | 2 X D + 6 X P | 3941 | 832 | 146 | 70 | 60 | — |
| ST-008 gp120 monomer | This study | 14 weeks | 3 X D + 1 X P | 6091 | 733 | 267 | 89 | — | 339 |
|  | | | | | | | | | |
| *P* values** | ST-008 (14 weeks) vs. SOS-IP (20 weeks) | | | 0.005 | 0.025 | <0.001 | <0.001 | — | — |
|  | ST-008 (14 weeks) vs. SOS-IP (54 weeks) | | | 0.260 | 0.735 | 0.021 | 0.302 | — | — |
| * D, DNA immunization using electroporation; P, protein boost  ** Two-tailed homoscedastic t-test using log_10_(IC50) with *n* = 4 for gp140 SOS-IP trimer and *n* = 8 for ST-008 | | | | | | | | | |
